# Managing a Department of Obstetrics and Gynecology in Times of COVID-19 Outbreak: The Foch Hospital Experience

**DOI:** 10.3389/fsurg.2021.564145

**Published:** 2021-04-13

**Authors:** Rouba Murtada, Marie Carbonnel, Aurélie Revaux, Angeline Favre-Inhofer, Jean-Marc Ayoubi

**Affiliations:** Department of Gynecology and Obstetrics, Foch Hospital, Suresnes, France

**Keywords:** COVID-19, reorganization, facemask, healthcare worker, patient safety, vaccination

## Abstract

Departments of Gynecology and Obstetrics, as other departments, were faced with a major challenge at the outbreak of the COVID-19 pandemic. Fast restructuring was necessary in order to provide the means for COVID-related care. In this article we share our 1-year experience in reshaping our activities, managing healthcare workers and securing a pathway for pregnant patients, including potential, and confirmed COVID-19 cases. Priorities were set on ensuring patients' and healthcare workers' safety. Key containment measures included facemasks, systematic screening, dedicated spaces for COVID-19 cases with reinforced measures and vaccination campaign.

## Introduction

The COVID-19 pandemic has taken the global French hospital system by surprise, forcing to restructure in an unprecedentedly rapid manner. This process has unveiled a certain degree of unpreparedness, with a lack of readily available procedures for such a situation, and the finite nature of resources: masks, tests, and manpower. Ultimately hard work and resilience prevailed.

Almost all departments had to revisit their daily activities, reducing some, and plainly canceling others, deemed non-urgent procedures, for the benefit of COVID-19 related care. The department of Obstetrics and Gynecology, dedicated to women's health in all of its aspects, was no exception (1). This was however a dual challenge for both maintaining a safe circuit for pregnant women, as they in the 3rd trimester of their pregnancy are more at risk of developing severe forms of the disease (2), and reducing or canceling other non-urgent activities.

In this article we share our experience in adapting our obstetrical and gynecological care to the sudden COVID-19 crisis, the evolutions performed during 1 year and the current issues. The emphasis has been put on managing medical, paramedical staff and spaces. We focused on organizational issues and looking at one primary parameter, COVID transmission among health care workers.

Our department is part of the Foch University Hospital in the Paris suburban area. Like many Obstetrics and Gynecology departments, it is a comprehensive structure. Obstetrics comprises a labor ward with 3,500 births per year, a post-partum and an antenatal ward. Gynecology manages its own procedures and all post-surgical care, emergency cases and pregnancy emergencies occurring before 23 weeks of gestation.

Awareness was raised about the issue of COVID-19 in the course of February 2020. Measures were taken progressively as the urgency of dealing with the contagiousness of COVID-19 and the advancing epidemic on the hospital level grew. In 2021, while vaccination is starting, tension within the hospital remains high. Indeed, the arrival of more contagious and potentially more serious variants of the virus is leading to a strengthening of our measures (3).

The four main axis of action are as follows:

1. Coping with new imperatives: wearing disposable masks for all

It has become increasingly clear that facemasks worn at all times is a cornerstone in the effort to limit disease spread, whether at the level of the general population in public settings, or in the hospital community.

The rationale lies in the high incidence of asymptomatic and pre-symptomatic transmission, which is a particularity of SARS-COV-2. One of its early demonstrations was the number of asymptomatic cases noted aboard the Diamond Princess cruise ship, estimated at a proportion 17.9% (4). It was again assessed in a nursing facility, when systematic RT-PCR testing in all residents following the identification of COVID-19 resulted in 56% of asymptomatic individuals at the time of testing (5). Equally important, is the observation that the viral load was similar in symptomatic, asymptomatic and pre-symptomatic patients (5).

The active replication of the virus in the upper respiratory tract and the latency with the apparition of symptoms (6), justify a strict containment policy which cannot be solely based on symptom-oriented screening.

In this setting, social distancing and elective wearing of facemasks only in symptomatic patients was an insufficient barrier to transmission. Conversely realizing that asymptomatic persons can be contagious led to the universal wearing of masks. Initially this was limited by a severe shortage of masks in France. Hence, on March 17, at the onset of the outbreak the use of masks was restricted to departments considered to be at high risk of exposure. This included intensive care units (ICUs) and emergency departments. On March 20, the use of masks became generalized to all departments including ours. All healthcare workers and patients were advised to wear a surgical mask at all times, especially when in contact with other individuals.

Without a high level of evidence, studies have suggested FFP2/N95 respiratory masks do not offer better protection than regular surgical masks in non-aerosolizing situations of health care (7). This combined with the fact that N95 masks were in short supply has led the French Society for Hospital Hygiene to recommend their use only when performing invasive medical procedures or procedures on the respiratory sphere (8). In accordance with the above, we equipped all healthcare workers (HCW) and patients with surgical masks, and dedicated N95 masks to HCWs having close contact with COVID-19 patients or suspicious of. During labor and especially during delivery, effective face-masking for patients was impossible. HCWs had to wear a supplementary visor.

2. Reorganizing activities in pandemic time

The cancellation of all non-urgent and non-essential activities, both medical and surgical, was instructed by public authorities (namely the Regional Health Authority-Agence Régionale de la Santé) during the first lockdown (March–May 2020). It came both as a response to a pressing need for COVID-dedicated critical care and in preparation for a potential wave of ICU-requiring patients. The intended effect of shutting down non-essential procedures was to free up resources whether structural (operating theater, post-interventional units) or manpower (nurses, doctors) for COVID-related tasks. The collateral effect was reinforcing the lockdown for the general population, avoiding patient-HCW contamination and compensating for COVID-related absenteeism, whether in real-time or anticipated.

The department of reproductive medicine was closed following a decision taken by the Agency of Biomedicine, which oversees all fertility care in France. All consultations were initially canceled, and all new treatments were ceased. Fertility preservation procedures for women including for cancer patients were suspended.

All non-urgent gynecological care in-patient appointments were canceled, and with it all hysteroscopy and ultrasound scan activities. All eligible activities were shifted to phone appointments and as adjustments were made and offices equipped with cameras, virtual appointments for telemedicine were initiated. It was important while contacting patients to advise of the shift to telehealth, to question about suspicious symptoms and screen for patients who require prompt physical examination.

Follow-up of pregnant women evolved toward an organized alternation of in-presence and virtual appointments, according to risk factors. In case of low-risk pregnancy, in-between appointments were virtual except for the 36 and 39 gestational weeks visits. This was to be corroborated by recommendations emanating from the national OBGYN college (CNGOF) on April 1, which were called “quick answers” to the crisis (9).

Waiting rooms were adjusted in order to allow sufficient space between patients. All patients had to wear a facemask. Hydro-alcoholic hand rub gel was supplied and to be used regularly before during and after the visit. Starting March 19, no accompanying person was allowed. A room was designated for COVID affected patients.

Regarding surgical activity, all but selected oncology procedures were initially canceled. Elective procedures were maintained in patients with time-sensitive cancer conditions, no suspicion of COVID and after reassessment of benefit/risk ratio depending on age and medical history. As the peak of ICU cases decreased in our facility, freeing up resources, we included cases of pre-invasive cancer and painful conditions. It was of strategic importance to keep a log of all canceled procedures to anticipate rescheduling in the post-crisis period, with color-coding according to degree of urgency.

After the first lockdown, 50% of surgery and IVF procedures were post-poned from May to early September 2020. Decreases in hospitalizations and improvements in the management of COVID-19 patients allowed to return to normal surgical and IVF activity after this date. Teleconsultations were mixed with face-to-face consultations to allow social distancing in waiting rooms.

3. Systematic testing and dedicated channels for COVID patients

First, a systematic screening with RT-PCR testing was not available. We created a register for suspicious cases amongst patients with symptoms. Thirty percent of them had a positive RT-PCR testing (*n* = 15/46). Then, we performed systematically for all hospitalized patients a RT-PCR in order to also identify asymptomatic patients. During the “second Covid wave” (November 2020) almost 1/10 women had a positive RT-PCR at delivery, mainly asymptomatic.

The French health authorities represented by the Regional Health Agency were clear in their recommendations about the necessity to create two separate COVID positive and COVID negative tracks and units (10). However, the particular setting of the obstetric population, whereby fetal/neonatal surveillance is additionally needed, prevented from admitting patients in the adult medicine dedicated COVID units. This was especially true in non-ICU requiring patients. Dedicating a special COVID unit for pregnant and post-partum patients implied an entirely separate roster for senior doctors, residents, nurses and childcare assistants. Moreover, anticipating the post-crisis period, in such a setting it would be made of overstaffed COVID units and understaffed non-COVID units. Finally, the time for diagnosis initially being of 48 h with the RT-PCR assay performed in our institution, a number of patients could be allotted to the wrong unit in the interval. For these reasons, we preferred dedicating designated rooms for COVID patients and PUIs within the different units.

In the labor and delivery ward, 2 suites were designated for COVID patients. In case of cesarean sections there was a designated operating room (OR), where the 2-h post-interventional surveillance also took place instead of moving the patient to the usual separate post-surgical recovery unit. An additional midwife consolidated the team for COVID-related activities, and an entire team (doctor, midwife, nurse, childcare assistant) was on-call. As much as possible and within reasonable limits, obstetrical activities were scheduled for COVID patients during the first lockdown (March–May 2020). A repeat cesarean section was scheduled on the day of the induction of labor, in a multipara at 41 weeks of gestation. This reduced the number of identified COVID pregnant patients near term and likely to present at an unpredicted time.

Four rooms within the gynecology ward were dedicated to either suspicious or confirmed COVID pregnant and post-partum patients. The rooms were intentionally designated outside of the antenatal and post-partum wards. Geographically they were located in a cul-de-sac, therefore an area with less active passage. The two adjacent rooms could also be turned into COVID specific rooms if needed. A designated nurse consolidated the team when needed and was dedicated to COVID patients.

Standard, contact and airborne precautions were applied with personal protective equipment (PPE) available in front of each room. Disposable material was privileged. Pre-prepared sets of material for various types of interventions were available in each room, avoiding comings and goings to procure material.

As in the rest of the hospital, all outside visits were canceled during the first lockdown. For the delivery itself, partners were present only during active labor and delivery, for 2 h post-partum and upon discharge. In order to provide better support for mothers in the post-partum ward and compensate for the partners' absence, the interventions of lactation consultants were increased. After May 2020, Visits and particularly that of partners were allowed but restricted according to epidemiological data.

4. Managing the team of healthcare workers

The cancellation of afore mentioned activities fulfilled its purpose of allowing the redeployment of HCWs where most needed, and strengthening the core of the taskforce dedicated to COVID patients during the first French lockdown.

However, at the outbreak of the epidemic, the crux of the matter was satisfying the surge in need for HCWs when it seemed many were going to be missing. Indeed, within the first 15 days of the lockdown, from mid-March to early April, ~20 HCWs took a leave of absence. In the labor and post-partum ward, out of 53 midwives there were 13 on sick leaves and out of 19 nurses, 4 sick leaves, all between the 15th of March and 11th of April. In the gynecology ward, there were 3 sick leaves, 2 for confirmed COVID, the last on the 30th March. Out of 7 head nurses, 5 were COVID positive.

Not all sick leaves were for illness, as 8 workers were furloughed due to underlying health conditions affecting them or their child or partner, putting at a high risk of a severe form of disease. Similarly, there were 2 sick leaves due to pregnancy, the first because of COVID illness, and the other pre-emptively. This attitude was consistent with the RCOG's recommendation that healthcare workers more than 28 weeks pregnant avoid patient contact (11). Most cases were noted in the beginning of the epidemic, before masks were routinely used, which therefore strongly advocates the protective role of facemasks in preventing further disease spread. However, this was not an isolated measure as most staff meetings were canceled. The only meetings maintained were the oncology multidisciplinary team meetings and the morning staff meeting for obstetrics with minimal attendance.

Vaccination became available in January 2021 at our hospital: HCW aged more than 50 years or with high risks comorbidities benefited from the RNA vaccine Pfizer-BioNTech. The Oxford-AstraZeneca vaccine became available in February for younger HCW. The incidence of reported adverse effects led many HCW to shun away from the vaccination.

## Discussion

For the first time in France in a century, health facilities are experiencing the true plight of a full-scale pandemic. While the SARS epidemic in 2003 and Ebola outbreak in 2014 were fair warnings, the death toll remained globally low. Unfortunately, the containment measures exerted previously have not sufficed for COVID-19, due to multiple factors, some inherent to the virus, others to human behavior and global changes (12).

Foch Hospital and our department had to undergo profound changes to tend to the pressing needs of COVID ICU-care. It was mainly the surgical activity and outpatient clinic that had to be idled down in order to dispatch the necessary taskforce for the COVID emergency as recommended by scholarly organizations (1, 13). For obstetrical care, however, no reduction of activity could be implemented and full staff remained necessary.

While controlling this communicable disease is of utmost importance, the long-term effect on individuals is significant. COVID 19 has negatively impacted patients with other diseases due to canceled care (14). That's why, we tried as soon as possible to restore a normal activity following the first lockdown. Tele consultation, installed quickly in our unit, was an effective way to maintain heath care for suitable indications and avoid COVID 19 transmission.

A high frequency of maternal mental health problems, such as clinically relevant anxiety and depression, during the epidemic are reported in many countries (15). Anxiety could be due to fear of own complications, Intrauterine, breastmilk transmission, and the passage of the virus to baby during and after delivery even if data are reassuring (2). The guidelines for labor, delivery, and breastfeeding for COVID-19 positive patients vary, and could create uncertainty and unnecessary harm. Modifications in general health care policies implemented due to COVID and notably, the restrain imposed on visits in the hospital, including that of the partner, strengthened the feeling of isolation experienced by many patients.

We have maintained the presence of the partner during labor even during the early outbreak, essential for patients support. More focus on maternal mental health during the epidemic should be enphasized.

In dealing with the crisis, efforts were centered around preventing disease spread and ensuring safety for patients as well as for healthcare workers. This was achieved through simple measures: universal facemasks, appropriate PPE, proper distancing, limitation of visits, dedicated rooms. These measures are to be applied at all times, including when not in contact with patients. Inter-colleague contamination played a part in a number of sick leaves in our department. Kabesch et al. experienced a similar but more important wave of COVID cases among their staff, due in part to meetings that took place amongst colleagues outside of the workplace (16). They were able to contain transmission through simple measures, mainly facemasks, testing and tracing index subjects. Emergence of more transmissible variants could require use of more protective respiratory masks: FFP2/N95 but data are still lacking.

It appears that it was not so much the number of COVID patients and PUIs that was time consuming. The strict and lengthy procedures for donning and doffing PPE, repeated several times a day, were the main reason for the need to have increased staffing. The first effect of an excessive load of work could be less rigor in the use of PPE, and an increased risk of self and cross-contamination.

During the SARS epidemic, the risk of colonization of HCWs even with proper PPE was found to be 11% in a study by Ho et al. (17). This could be explained by mistakes in using PPE, and stresses the need for training.

Personal protective equipment was particularly important in the delivery room when assisting a vaginal delivery, given the pushing efforts required. Vaginal delivery is highly aerosolizing, so it is safer to reinforce protective measures (18). In two publications regarding management of pregnant women during the COVID pandemic, Capanna et al. and Stephens et al. also considered pushing efforts as at high risk for disease transmission through respiratory secretions (19, 20).

Vaccination is a great hope to beat COVID-19 pandemic. HCW are the highest priority recipients of the vaccination. In principle, HCW should the first to receive vaccination. Yet, due to worldwide shortages not all HCW can be vaccinated in a timely manner. Efficacy and safety of RNA vaccine and Oxford-Astra Zeneca for people under 65 years has been proven (21, 22). Emergence of variants could decrease their efficacy, but data are mandatory. Studies about RNA Sars-Cov2 vaccines and Pregnant women are lacking, but preliminary data are reassuring (23).

## Conclusion

The contagiousness of SARS-COV-2 and especially the higher possibility of asymptomatic transmission constitute a true problem for the health care management. The need to separate affected and spared individuals, whether patients or healthcare workers, is still valid. To distinguish between a healthy individual and possible asymptomatic carrier is difficult in practice. Every subject must be considered as potentially infected. Basic but efficient protective measures such as facemasks, PPE and designated rooms associated with systematic screening have been instrumental in containing the disease spread. These are as essential in patient to HCW interactions as they are amongst HCWs, in order to safeguard the workforce and ensure patient security. Vaccines, which became recently available, offer a great hope to soon circumscribe the COVID-19 pandemic.

## Data Availability Statement

The original contributions presented in the study are included in the article/supplementary material, further inquiries can be directed to the corresponding author.

## Author Contributions

J-MA conceived the idea for the content of the article. RM drafted the original manuscript. MC and AR critically revised the manuscript and contributed valuable feedback. AF-I gathered and provided data for the drafting of the manuscript. All authors contributed to the article and approved the submitted version.

## Conflict of Interest

The authors declare that the research was conducted in the absence of any commercial or financial relationships that could be construed as a potential conflict of interest.
